# Cognitive performance and leukocyte telomere length in two narrow age-range cohorts: a population study

**DOI:** 10.1186/1471-2318-10-62

**Published:** 2010-09-16

**Authors:** Karen A Mather, Anthony F Jorm, Kaarin J Anstey, Peter J Milburn, Simon Easteal, Helen Christensen

**Affiliations:** 1 Centre for Mental Health Research, Australian National University, Canberra, ACT, 0200, Australia; 2 Brain and Ageing Research Program, School of Psychiatry, University of New South Wales, Sydney, NSW, 2031, Australia; 3 Orygen Research Centre, University of Melbourne, Melbourne, Vic, 3052, Australia; 4 John Curtin School of Medical Research, Australian National University, Canberra, ACT, 0200, Australia

## Abstract

**Background:**

Cognitive function and telomere length both decline with age. A correlation between these two measures would suggest that they may be influenced by the same underlying age-related biological process. Several studies suggest telomere length may be positively correlated with cognitive performance but the evidence is equivocal. In this report, the relationships between telomere length and cognitive performance at Wave 2 and cognitive change from Wave 1 to Wave 2 are assessed in two narrow age-range population cohorts.

**Methods:**

We tested the hypothesis that leukocyte telomere length correlates positively with cognitive performance and cognitive decline in two community cohorts of middle-aged (*n *= 351, 44-49 years) and older (*n *= 295, 64-70 years) adults, who participated in two waves of a longitudinal study undertaken in the Canberra-Queanbeyan region of Australia. Telomere length was estimated at Wave 2. Cognitive performance was measured using the Symbol Digit Modalities Test, the immediate recall test of the California Verbal Learning Test, reaction time (simple & choice) and the Trails Test Part B.

**Results:**

Cross-sectionally at Wave 2, telomere length correlated with Symbol Digit Modalities Test scores (men) and simple reaction time (women) for the older cohort only, although the latter finding was in the opposite direction to that hypothesised. Telomere length measured at Wave 2 was not associated with cognitive change from Wave 1 to Wave 2 for either cohort, except for two associations of small magnitude (immediate recall in the older cohort, choice reaction time in older women), which were also in the contrary direction to that predicted.

**Conclusions:**

These results do not give strong support to the hypothesis that leukocyte telomere length is associated with either levels of cognitive performance or age-related cognitive change.

## Background

Telomeres are DNA-protein structures that form protective caps at the termini of chromosomes and contribute to genomic stability. The length of telomeres decreases with increasing age [1-6], as a result of processes such as the incomplete replication of the chromosomal ends at each cell division [5] and during periods of elevated oxidative stress [7-11].

Cognitive decline also accompanies the ageing process [12-14] and therefore may be correlated with telomere length. Few studies have examined this relationship. Three recent reports investigated cognitive performance and leukocyte telomere length using population samples, with mixed results. Harris et al. [15] found a single inverse correlation between a verbal fluency test and telomere length, but did not observe any significant relationships with tests of non-verbal reasoning, verbal declarative memory and a dementia-screening test in a sample of 79 year olds. In contrast, Valdes et al. [6], using a larger and younger female sample with a wide age range (*mean *age 50 yrs, range 19-78 yrs), found better performance was correlated with longer telomere length on four of six cognitive tests (simple reaction time, space span test, paired associate learning and delayed matching to sample), but not with pattern recognition and spatial working memory tasks. The most robust findings were observed for the space span and the delayed matching to sample tests (partial *r *= 0.16), which remained significant after adjustments for a wide range of variables. Whether these results also apply to men is unclear. In the most recent study, Yaffe et al. [16] observed that longer baseline telomere length was associated with better baseline performance on the Digit Symbol Substitution Test (DSST), but not for change scores over seven years, in a large sample of 70 to 79 year olds. In addition, a number of investigators have examined relationships with telomere length using broader cognitive status questionnaires, such as the Mini-Mental State Examination [MMSE, [17]]. The MMSE has ceiling effects, which reduces its utility in younger samples and in general these studies have found negative results [15,18,19]. One study examined change in MMSE scores over three years, but found no relationship with baseline telomere length in a sample aged 85 and over [19]. In contrast to these longitudinal results, Yaffe et al. [16] observed that shorter baseline telomere length was associated with greater decline in performance on the Modified MMSE over seven years, but not with baseline scores.

There are a number of methodological issues that need to be considered when interpreting these findings. The age and the age range of the sample employed in such studies may be critical. The use of a sample with a wide age-range may inflate observed relationships due to age-related mean trends [20,21]. Therefore, cross-sectional studies using a wide age range may not provide the optimal design for evaluating the inter-relationships between age-sensitive measures. Conversely, the results of studies that employ a sample of a specific age may not be generally applicable, as these relationships may change across the lifespan. Sequential narrow age-range cohorts or longitudinal designs are more appropriate for clarifying such relationships. Longitudinal data would allow the examination of individual rates of biological ageing with changes in telomere length over successive waves of data collection. However, limited longitudinal data is available. Thus, more research is required to address this question using both men and women and to examine these relationships at different stages of the lifespan, whilst controlling for premorbid intelligence and other potential confounding measures.

The present study examined the relationships between telomere length and cognitive performance across a range of tests in two narrow age-range cohorts - individuals aged in their 40s and 60s. Telomere length was estimated in peripheral blood leukocytes at the second wave of an ongoing longitudinal study. At Wave 2, we predicted positive correlations between telomere length and cognitive performance and that these relationships would be stronger in the older age cohort, since both cognitive performance and telomere length decline with age. As we had baseline cognitive performance data from four years earlier, we were able to correlate telomere length at Wave 2 with measures of cognitive decline. We predicted that those with shorter telomeres would have greater cognitive decline over the preceding four years.

## Methods

### Participants

**Figure 1:**
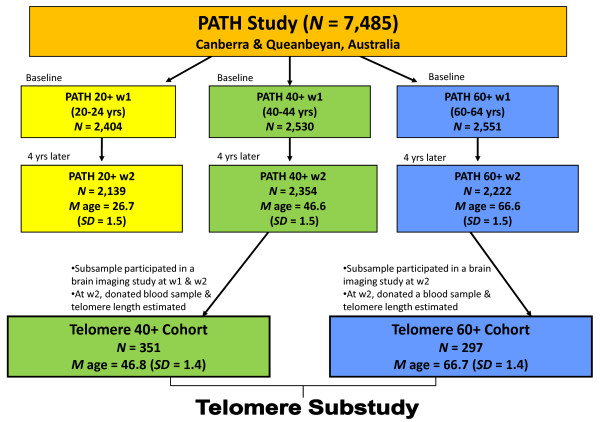
**The relationships between the 40+ and 60+ Telomere Cohorts and the PATH Through Life (PATH) Study**. The flow chart shows the recruitment of the 40+ and 60+ Telomere Cohorts from the PATH Study. Telomere length was estimated at Wave 2 and cognitive performance was measured at Waves 1 (w1) and 2 (w2) (except for the Trails B Test, which was measured at w2 only).

Participants were drawn from a large longitudinal Australian population-based study, the PATH Through Life Project (PATH), which began in 1999 and collects data every four years. This project investigates the health and well-being of PATH participants by collecting self-report data and assessing cognitive, physical and health measures. The PATH study is comprised of three narrow age-range cohorts and participants were aged 20-24, 40-44 (40+) and 60-64 (60+) years when recruited into the study at Wave 1. The PATH sample was recruited using the compulsory Australian electoral roll records in the Queanbeyan and Canberra regions. The majority of the interviews occurred in the participant's home and participants entered self-report data using hand-held computers under the supervision of trained interviewers who also administered the physical and cognitive tests. The Human Research Ethics Committee of the Australian National University gave approval for this study and all participants gave informed consent. Further details of the study are provided in Jorm et al. [22]. When the entire PATH sample was compared with census information from the recruitment areas, participants were similar in marital status but tended to have greater rates of employment and higher education levels [23]. Telomere length was measured in a subsample of the 40+ and 60+ cohorts who provided a blood sample for genetic analyses at the second wave of data collection. A flow chart showing the relationships between the telomere cohorts and the PATH Study is illustrated in Figure 1. The recruitment of the telomere cohorts is described in more detail below.

#### Telomere 40+ Cohort

Over 25% of the 40+ cohort (*n *= 656) were invited to participate in a brain imaging sub-study at Wave 2. Most of the invited PATH participants expressed an interest in participating (*n *= 503), with 372 donating a blood sample. Telomere length data was available for 351 participants with a mean age of 47 years (*SD *= 1.4). Compared to the remainder of the 40+ PATH cohort, the 40+ telomere cohort were somewhat older but did not differ in the proportions of sex, marital status, number of years of education, depression scale [24] or Symbol Digit Modalities Test [25] results.

#### Telomere 60+ Cohort

All PATH 60+ cohort participants were invited to participate in a longitudinal brain imaging sub-study at Wave 1. More than 80% indicated a willingness to participate (*n *= 2076) and 479 participated at Wave 1. At Wave 2, 297 participants provided a blood sample before the commencement of this study. Telomere length data was available for the 297 participants, two of which were identified as extreme outliers and omitted from further analyses (z-scores > 3.29) [26]. The 60+ telomere cohort had a mean age of 66.7 years (*SD *= 1.4) and did not differ from the remainder of the 60+ PATH cohort with respect to age, sex, years of education, the Mini-Mental State Examination [17] or depression scale scores. However, the 60+ telomere cohort performed slightly better on the Symbol Digit Modalities Test.

### Telomere length measurement

Standard procedures were used to extract DNA from peripheral blood leukocytes and positive control samples [for further details, see [27]]. The positive controls consisted of saliva and cell line genomic DNA and low and high molecular weight telomere genomic DNA standards (TeloTAGGG Telomere Length Assay Kit, #2 209 136, Roche, Basel, Switzerland).

Estimation of telomere length was performed using a quantitative real-time PCR (Q-PCR) technique [28]. In this method, two Q-PCRs were undertaken for each sample, a single-copy gene-specific Q-PCR (acidic ribosomal phosphoprotein, *36B4 or RPLP0*) and a telomere-specific Q-PCR. A telomere/single-copy gene (T/S) ratio was calculated for each sample using the Q-PCR results and normalised to the T/S ratio of a reference sample. The normalised T/S ratio was used as an estimate of relative telomere length.

Estimation of telomere length was performed separately for each age cohort. The telomere and 36B4 Q-PCR assays were performed in separate plates. Quadruplicate replicates of each DNA sample (5-10 ng/reaction) were dispensed using a liquid handling robot (EpMotion 5070, Eppendorf, Hamburg, Germany) into duplicate plates and dried down. Replicates were placed together in matching positions on the duplicate plates. Dehydrated DNA was resuspended in the relevant Q-PCR reaction mix, containing the appropriate primers, 1× Master Mix (Platinum SYBR Green Master Mix, #11744-500, Invitrogen, Carlsbad, CA, USA) and H_2_O to a total volume of 10 μl. For the telomere Q-PCR reaction, the final concentration of each of the primers was 500 nM (Tel1b: 5' CGG TTT GTT TGG GTT TGG GTT TGG GTT TGG GTT TGG GTT 3'; Tel2b: 5' GGC TTG CCT TAC CCT TAC CCT TAC CCT TAC CCT TAC CCT 3'; Cawthon, 2003, pers. comm.). The final concentrations of the primers for the 36b4 Q-PCR reaction were 390 nM of the 36b4u primer and 650 nM of the 36b4d primer [36b4u: 5' CAG CAA GTG GGA AGG TGT AAT CC 3'; 36b4d: 5' CCC ATT CTA TCA TCA ACG GGT ACA A 3', [28]]. The Q-PCR assays were performed in a 7900HT Fast Real-Time PCR machine (Applied Biosystems, Foster City, USA) at the ACRF Biomolecular Research Facility (Australian National University) using the cycling program of 50°C/2 min, 95°C/10 min, followed by 30 cycles of 95°C/30 secs and 56°C/1 min.

Q-PCR results were screened for outliers if the *SD *of the cycle threshold [Ct] was greater than 0.2 [29]. If the removal of a single outlier did not improve the *SD *of the replicate Cts for a sample (≤0.2), the assay was repeated or the sample omitted. The variability of the Q-PCR results was assessed for a set of positive controls (human cell line and saliva DNA, *n *= 4 for the 40+ cohort and *n *= 3 for the 60+ cohort) that were assayed in quadruplicate across five plates. For the 40+ cohort, the mean coefficients of variation (CVs) for Ct scores were 0.43% and 0.76% for the single-copy-gene and telomere Q-PCR assays respectively. For the 60+ cohort, the average CVs for Ct scores were 0.53% and 0.62% for the single-copy-gene and telomere Q-PCRs respectively.

Relative telomere length (normalised T/S ratio) was calculated using the comparative Ct method after verifying that the telomere and 36B4 Q-PCRs had equivalent amplification efficiencies [30]. Mean inter-assay CVs for the relative telomere estimates (normalised T/S ratio) across assays for the positive controls were 8.82% and 3.44% for the 40+ and 60+ cohorts respectively.

The relative telomere length results were validated using an alternate method, the telomere restriction fragment method (TRF) using the TeloTAGGG Telomere Length Assay Kit (Roche) for a set of positive control samples. In contrast to the Q-PCR method, the TRF method estimates telomere length in kilobases. The two methods were highly correlated for each age cohort (*r *> 0.9, *p *< .03, one-tailed). The regression equation describing the relationship between the two methods was also used to convert the relative telomere length estimate to an absolute kilobase measure for each age cohort. The kilobase estimate was used to assess if there was a significant telomere length difference between the two age cohorts. All primary analyses used the relative telomere length measure.

### Measures

#### Cognitive Measures

Information processing speed and attention were evaluated using the Symbol Digit Modalities Test (SDMT) [25]. Episodic memory (immediate recall) was assessed using the first trial of the California Verbal Learning Test [31]. Simple and choice reaction time were tested using a small hand-held box with two red lights and two depressible buttons located at the top of the box [32]. For the simple reaction time test, one of the red lights was activated and the participant was asked to push the right hand button immediately. In the choice reaction time test, the left or right red light was activated and the participant was asked to press the appropriate button. Simple reaction time was measured first, using four blocks of 20 trials, followed by two blocks of 20 trials measuring choice reaction time. Any values less than 0.1 secs or greater than 3.0 seconds were removed. For each participant, means were then calculated for each block and any values that exceeded 3 standard deviations were eliminated [32]. The means (secs) were then re-calculated. Executive function was assessed using the Trails Test Part B [33]. Spot-the-Word Version A [34] was used as a test of pre-morbid intelligence. The Mini-Mental State Examination [17], a screening test for cognitive impairment/dementia, was administered to the 60+ cohort only. Whether telomere length at Wave 2 was associated with decline in cognitive performance from Wave 1 to Wave 2 was undertaken by assessing cognitive performance at Wave 2 residualised for Wave 1 scores. This analysis was not undertaken for the Trails Test as this was not administered at Wave 1.

#### Other Measures

Sociodemographic, lifestyle, health and anthropometric information were self-reported. Occupational social class (socioeconomic status) was determined using the Australian Standard Classification of Occupations [35] and participants were classified into one of three categories, professional, white collar and blue collar. Respondents were classified into three tobacco smoking categories: current, past or never [36]. Weekly alcohol consumption was estimated from responses to the Alcohol Use Disorders Identification Test (AUDIT) [37], taking into account binge drinking episodes [23,38]. Antioxidant vitamin use was defined as the consumption of vitamins and/or multivitamins 6-7 days/week for 6 months or more. Physical activity was measured using questions from the UK Whitehall II Study [39,40] to calculate the total number of hours of physical activity/week [41]. The Goldberg Depression Scale [24] was used to assess depressive symptoms and the current use of anti-hypertensive medication was recorded. Two blood pressure measurements were taken whilst the participant was sitting and the mean used for analyses. A measure of lung function, the forced expiratory volume in the first second (FEV1), was determined using a Micro Spirometer (Micro Medical Ltd, Rochester, Kent, UK). The highest of three FEV1 measurements was used in analyses. Using the dominant hand, two trials of handgrip strength were measured using the dynamometer (Smedley's Dynamo Meter, Tokyo) and the mean recorded.

### Statistical Analyses

The relative telomere length variable was log_10 _transformed to improve the normality of the distribution. Similarly, alcohol consumption, physical activity, body mass index (kg/m^2^) and the cognitive measures of reaction time (simple and choice) and the Trails Test were also log_10 _transformed. Missing data were minimal except for the measure of physical activity, where data was imputed. Missing values for physical activity were replaced with the median for the entire PATH Cohort when stratified by age group for each frequency of activity category except for '*never/hardly ever' *when the missing value was replaced with zero. The relevant analyses were adjusted for the telomere length covariates, alcohol consumption, physical activity and systolic blood pressure and for age, sex and pre-morbid intelligence using Spot-the-Word scores.

Most of the analyses were undertaken within each age cohort. Due to sex differences on most measures, the analyses were also undertaken separately for men and women. Simple bivariate and partial correlations (Pearson's and non-parametric) were undertaken to assess the relationships between continuous variables. Independent *t-*tests, analysis of variance (ANOVA) or χ^2 ^tests were used to assess the differences between groups. Generalized linear models were used to assess the association between telomere length at Wave 2 and cognitive change from Wave 1 to Wave 2. In order to interpret the size of the effects, telomere length and the dependent variables were converted to z-scores. Statistical tests were performed using SPSS v17 (SPSS Inc., Chicago, IL, USA) and a *p *value of < 0.05 was considered significant.

## Results

Descriptive statistics for the two age cohorts are given in Table 1. Fewer women were present in the 60+ cohort compared to the 40+ cohort (46.4% vs. 55.8%). There were more current smokers in the younger cohort and the older cohort undertook more hours of physical activity. Compared to the younger cohort, more 60+ cohort individuals consumed daily antioxidant vitamins and a higher proportion was hypertensive. There was a significant difference in telomere length between the two age cohorts with the 40+ cohort having longer telomeres (median = 5.65 kb, IQR = 5.15-6.52) than the 60+ cohort (median = 4.69 kb, IQR = 4.42-5.03) (*p *< 0.0001, equal variances not assumed). Age was not correlated with telomere length within each of the narrow age-range cohorts (*p *> 0.05).

**Table 1:** General characteristics by age cohort

Characteristic		40+ (*n *= 351)	60+ (*n *= 295)
Telomere Length (kb)		5.65 [5.15-6.52]^a ^ 5.88 ± 0.98^b^	4.69 [4.42-5.03] ^a ^ 4.74 ± 0.47^b^
Sociodemographic			
Sex	Men	155 (44.2%)^c^	158 (53.6%)^c^
	Women	196 (55.8%)^c^	137 (46.4%)^c^
Age (yr)		46.8 ± 1.4^b^	66.7 ± 1.4^b^
Age range (yr)		44-49	64-70
Education (yr)		14.80 ± 2.25^b^	14.10 ± 2.62^b^
Lifestyle			
Smoking Status	Current	45 (12.8%)^c^	17 (5.8%)^c^
	Past	117 (33.3%)^c^	107 (36.3%)^c^
	Never	189 (53.8%)^c^	171 (58.0%)^c^
Alcohol (drinks/week)		4.00 [1.00-9.00]^a^	4.00 [0.38-9.00]^a^
Physical activity (hr)/week		10.50 [5.50-17.00]^a^	14.00 [8.00-24.75]^a^
Daily antioxidant vitamin use		48 (13.7%)^c^	71 (24.1%)^c^
Health			
Obesity^d^		72 (21.8%)^c^	60 (20.5%)^c^
Definite hypertension^e^		46 (13.5%)^c^	133 (45.9%)^c^
Physical Function			
Grip Strength^f^		21.598 ± 6.081^b^	18.533 ± 5.153^b^
Lung Function^g^		1.859 ± 0.362^b^	1.513 ± 0.342^b^

kb = kilobase.

^a ^median [interquartile range].

^b ^mean ± standard deviation.

^c ^frequency (%).

^d ^Obesity is defined as a body mass index (kg/m^2^) of ≥30.00.

^e ^Hypertension is defined as either the current use of anti-hypertensive medication or having a mean systolic blood pressure measurement ≥ 160 mm Hg and/or a mean diastolic blood pressure ≥95 mm Hg.

^f ^Handgrip strength (kg) adjusted for height (m).

^g ^Lung function is the highest reading from three trials for forced expiratory volume (L) in one second (FEV1), adjusted for height (m). Those with lung conditions were excluded.

Potential covariates of telomere length identified from prior studies include sex [42-45], antioxidant vitamin use [46,47], physical activity [48], alcohol consumption [15], tobacco smoking [49,50] and socioeconomic status [51]. There were no significant differences in telomere length between men and women, nor for antioxidant vitamin use, smoking or socioeconomic status (*p *> 0.05). For the 60+ cohort only, increased alcohol consumption was correlated with shorter telomere length (*r *= -0.155, *p *= 0.008). Telomere length was also correlated with physical activity for the 40+ cohort (*r *= -0.114 *p *= 0.034) and systolic blood pressure for 40+ women (*r *= 0.157, *p *= 0.029). The results of these analyses are detailed in additional file 1 (Supplemental Table S1).

Cross-sectional analyses revealed no significant correlations between telomere length and cognitive performance for the 40+ cohort or for men or women of this cohort (Table 2). For the entire older cohort, telomere length was not associated with performance on any of the cognitive tests except for a positive correlation with the SDMT after adjusting for covariates (Spearman's partial *r *= 0.135, *p *= 0.025). When men and women were examined separately, performance on the SDMT remained positively correlated with telomere length for men of the 60+ cohort only; before (*r *= 0.173, *p *= 0.031) and after adjustment for covariates (Spearman's partial *r *= 0.193, *p *= 0.018). These relationships were in the direction expected, that is, better cognitive performance was associated with longer telomere length. Conversely, for 60+ cohort women, but not men, a significant positive correlation between telomere length and simple reaction time in the opposite direction to that expected was observed. This relationship was significant before (*r *= 0.185, *p *= 0.036) and after adjusting for covariates (Spearman's partial *r *= 0.218, *p *= 0.018).

**Table 2:** Cross-sectional correlations between telomere length and cognitive scores (Wave 2) by age cohort and sex

Cognitive Test	Age Cohort	*r*	*P-*value	Partial *r*	*P-*value
Immediate recall	40+	0.021	0.694	-0.019	0.728
	40+ Men	-0.026	0.747	-0.053	0.520
	40+ Women	0.037	0.609	0.001	0.985
	60+	-0.065	0.263	-0.108	0.073
	60+ Men	-0.038	0.636	-0.125	0.130
	60+ Women	-0.114	0.186	-0.094	0.303
SDMT^a^	40+	-0.013	0.809	-0.001	0.979
	40+ Men	-0.107	0.186	-0.111	0.176
	40+ Women	0.061	0.393	0.088	0.231
	60+	0.104	0.076	0.135	0.025*
	60+ Men	0.173	0.031*	0.193	0.018*
	60+ Women	0.050	0.563	0.087	0.340
Simple RT^b^	40+	-0.033	0.543	-0.014	0.795
(secs)	40+ Men	-0.060	0.467	0.016	0.849
	40+ Women	-0.064	0.381	-0.047	0.528
	60+	0.076	0.204	0.099	0.108
	60+ Men	-0.051	0.529	0.005	0.950
	60+ Women	0.185	0.036*	0.218	0.018*
Choice RT	40+	0.000	0.997	-0.009	0.871
(secs)	40+ Men	0.007	0.929	0.013	0.880
	40+ Women	-0.022	0.767	-0.025	0.732
	60+	0.017	0.770	0.036	0.556
	60+ Men	-0.121	0.134	-0.051	0.546
	60+ Women	0.139	0.114	0.136	0.143
Trails B	40+	0.045	0.398	0.036	0.506
	40+ Men	0.139	0.085	0.137	0.095
	40+ Women	-0.038	0.601	-0.063	0.388
	60+	-0.024	0.680	-0.025	0.674
	60+ Men	-0.095	0.239	-0.080	0.331
	60+ Women	0.036	0.678	0.017	0.852

TL = telomere length; SDMT = Symbol Digit Modalities Test; RT = reaction time;

*p < 0.05. Partial correlations were adjusted for age, Spot-the-Word, alcohol consumption, physical activity, systolic blood pressure and sex were appropriate.

^a ^For SDMT, outliers (*z*-scores > +3.29 or < -3.29) were omitted from the bivariate correlations for the 60+ cohort.

^b ^For simple reaction time, outliers (*z*-scores > +3.29 or < -3.29) were omitted from the bivariate correlations.

Cognitive performance test scores at the two waves of data collection (Waves 1 & 2) are shown in Table 3. Better cognitive performance was observed on most cognitive tests for the younger cohort. For the older cohort, cognitive performance decreased across the two waves of data collection whereas for the 40+ cohort, performance either slightly decreased or improved. As indicated in Table 3, in general, telomere length at Wave 2 was not associated with change in cognitive performance over the two waves of data collection (Wave 1 to Wave 2) in either cohort (*p *> 0.05). The exceptions were (i) a significant association between telomere length and immediate recall for the entire 60+ cohort (*p *= 0.049, B = -0.100, S.E. = 0.051) and (ii) a significant association with choice reaction time for women of the older cohort (*p *= 0.029, B = 0.169, S.E. = 0.078). However, the magnitude of the effects was small and in the contrary direction to that expected.

**Table 3:** Cognitive performance at the two waves of data collection (Wave 1 and Wave 2) for each telomere cohort

Cognitive Test	Age Cohort	*n *at Wave1	**Wave 1 Mean ± *SD***^***a ***^**or Median [*IQR*]**^**b**^	*n *at Wave 2	**Wave 2 Mean ± *SD***^***a ***^**or Median [*IQR*]**^**b**^	***P*-value**^**c**^
Immediate recall^d^	40+	350	8.21 ± 2.18*^a^*	351	8.33 ± 2.24*^a^*	0.694
	40+ Men	155	7.69 ± 1.97*^a^*	155	7.74 ± 2.05*^a^*	0.612
	40+ Women	195	8.63 ± 2.26*^a^*	196	8.80 ± 2.27*^a^*	0.665
	60+	295	7.51 ± 2.13*^a^*	293	7.16 ± 1.99*^a^*	0.049*
	60+ Men	158	7.11 ± 2.05*^a^*	157	6.86 ± 1.86*^a^*	0.307
	60+ Women	137	7.96 ± 2.14*^a^*	136	7.51 ± 2. 08*^a^*	0.102
SDMT^e^	40+	349	61.30 ± 8.79*^a^*	350	60.94 ± 8.59*^a^*	0.966
	40+ Men	155	60.70 ± 8.65*^a^*	155	60.45 ± 8.67*^a^*	0.442
	40+ Women	194	61.78 ± 8.89*^a^*	195	61.32 ± 8.54*^a^*	0.416
	60+	294	51.40 ± 8.00*^a^*	293	50.58 ± 8.23*^a^*	0.058
	60+ Men	158	51.42 ± 7.65*^a^*	156	50.93 ± 7.71*^a^*	0.059
	60+ Women	136	51.38 ± 8.41*^a^*	137	50.19 ± 8.79*^a^*	0.292
Simple RT	40+	342	0.224 [0.206-0.245]^b^	342	0.226 [0.210-0.250]^b^	0.833
	40+ Men	150	0.216 [0.200-0.234]^b^	150	0.223 [0.207-0.243]^b^	0.553
	40+ Women	192	0.228 [0.211-0.257]^b^	192	0.232 [0.213-0.260]^b^	0.390
	60+	285	0.238 [0.218-0.267]	285	0.252 [0.228-0.290]^b^	0..498
	60+ Men	150	0.234 [0.213-0.254]^b^	154	0.243 [0.223-0.285]^b^	0.354
	60+ Women	135	0.252 [0.221-0.281]^b^	131	0.260 [0.235-0.302]^b^	0.117
Choice RT	40+	338	0.283 [0.266-0.306]^b^	341	0.288 [0.263-0.310]^b^	0.700
	40+ Men	150	0.278 [0.262-0.300]^b^	149	0.278 [0.259-0.302]^b^	0.784
	40+ Women	188	0.287 [0.269-0.312]^b^	192	0.295 [0.265-0.316]^b^	0.615
	60+	283	0.314 [0.291-0.334]^b^	285	0.318 [0.288-0.349]^b^	0.068
	60+ Men	149	0.315 [0.289-0.330]^b^	154	0.317 [0.283-0.345]^b^	0.989
	60+ Women	134	0.313 [0.292-0.338]^b^	131	0.318 [0.294-0.354]^b^	0.029*

IQR = interquartile range; *SD *= standard deviation; Immediate recall is from the California Verbal Learning Test; SDMT = Symbol Digit Modalities Test; RT = reaction time; Descriptive statistics are given using the raw data. * *p *< 0.05

^a ^Mean ± standard deviation

^b ^Median [interquartile range]

^c ^Covariates were Wave1 performance on the relevant cognitive test, age, Spot-the-Word, systolic blood pressure, physical activity, alcohol consumption and where appropriate sex

^d ^For the immediate recall descriptives, outliers (*z*-scores > +3.29 or < -3.29) were omitted

^e ^For the SDMT descriptives, outliers (*z*-scores > +3.29 or < -3.29) were omitted

## Discussion

In general, telomere length was not associated with cognitive performance in two narrow age-range cohorts of middle-aged and older Australians. Although there was a significant correlation between performance on the Symbol Digit Modalities Test (SDMT) and telomere length in the expected direction, a significant association was also observed in the contrary direction for telomere length and simple reaction time. These relationships were modified by age group, with the significant findings being observed in the older age cohort only. Sex differences were also observed, with a significant correlation between telomere length and SDMT scores found for men only, whereas a significant association between telomere length and simple reaction time was observed for women only. Telomere length at Wave 2 was not correlated with cognitive change over a four-year period from Wave 1 to Wave 2, except for two associations in the older cohort (immediate recall and choice reaction time for women) of small effect size and in the contrary direction to that hypothesised.

Consistent with the present results, Yaffe et al. [16] observed a positive association between telomere length and a similar cognitive test to the SDMT, the DSST, but not with change scores, in a slightly older community sample. This suggests that leukocyte telomere length may be associated with cross-sectional performance on tests of information processing speed and attention in older samples. On the other hand, the generally negative results of the current study concur with those of Harris et al. [15] who in the main did not find any significant relationships between telomere length and a number of cognitive tests in an older sample of 79 year olds. The authors found one significant relationship between a test of executive function (verbal fluency) and telomere length in the opposite direction to that expected, which the authors speculate was attributable to chance. In the current study, a different test of executive function was employed, the Trails B Test, but no significant correlations with telomere length were observed in either age cohort. Valdes et al. [6] found a significant relationship between telomere length and simple reaction time in the expected direction, but this did not remain significant after controlling for a wide range of measures. Although the present study did find a significant relationship between telomere length and simple reaction time for older women, this was in the opposite direction to that predicted and observed by Valdes et al. [6]. Moreover, Valdes et al. reported that telomere length contributed 2.3% or less to the variance observed in cognitive ability. All of these studies vary in the samples used and, in general, different cognitive tests were employed.

Few studies have examined cognitive change and telomere length and the findings are equivocal [15,16,19]. Further longitudinal studies spread across the lifespan that use a wide variety of cognitive tests are required to adequately address this question.

In the current study, as expected, mean telomere length was significantly shorter for the older age cohort. Conversely, telomere length was not correlated with age within each age cohort, which may be due to the use of narrow age-range cohorts and the wide inter-individual variability observed in telomere length for individuals of the same chronological age [52]. The kilobase estimates of telomere length are similar to prior reports that have been calculated using the relative telomere length method [4,46,53]. The rate of telomere loss based on the difference between the two age cohorts (~50 base pairs/yr), is also congruent with other cross-sectional data [45,54-58].

There are limitations to the present study. Ideally, to examine the relationship between telomeres and cognition, telomere length should be measured in neural tissue such as neuronal or glial cells. However, access to such samples is not practical. Although telomere length in leukocytes may act as a surrogate marker for telomere length in other tissues [59-62], recent research suggests it may not be applicable to all cell types [63]. The current study was underpowered (54-75%) to detect correlations of similar size to the Valdes et al. [6] report; however, the study had sufficient power (> 80%) to detect correlations of medium to large effect size (*r *≥ 0.3) [64]. The variability of telomere length estimation as measured by the mean inter-assay coefficient of variation (CV) differed for the two cohorts. The larger measurement error for the 40+ cohort (8.8%) may have attenuated any significant relationships of small to medium effect size. Nevertheless, the inter-assay CV results of the present study fall within the range reported in prior studies, which is generally less than 10% but can range from 5.3 to 28.0% [65-68]. The comparatively low inter-assay CV for the 60+ cohort (3.4%) and the lack of significant relationships in general for this cohort, suggests that there are either no associations between the cognitive measures and telomere length or they are of small effect size. The minimisation of telomere length measurement error should be an important priority in future telomere studies. Another limitation is that longitudinal telomere length data is not available. Thus comparisons of the rates of telomere attrition with cognitive change were not possible. Further, not all possible telomere length covariates were measured, such as paternal age at the participant's birth [69].

The main strength of this study is the use of two narrow age-range cohorts that differ in age by twenty years allowing the examination of the relationships between cognition and telomere length to be investigated at two different stages of the lifespan. It also examined the relationships between cross-sectional telomere length and longitudinal performance on age-sensitive cognitive tests over four years. In the future, as further waves of cognitive data are collected, it will allow the examination of the relationships between prospective cognitive decline measures over a longer time period with 'baseline' telomere length.

## Conclusions

The results of the current study do not lend support to the hypothesis that leukocyte telomere length is associated with general cognitive performance or age-related cognitive change. Whether telomere length is a marker of cognitive ageing in the general population requires more data from a comprehensive range of cognitive tests, particularly from longitudinal and/or sequential narrow age-range cohort study designs.

## Competing interests

The authors declare that they have no competing interests.

## Authors' contributions

HC, AFJ, KJA and SE contributed to the concept and design of the study and to the interpretation of the results and helped write the manuscript. In addition, AFJ, KJA and HC participated in the design of the PATH Study and recruitment of participants. PM assisted with the telomere length measurements. KAM designed the study, performed the telomere length estimations, analysed the data and wrote the manuscript. All authors approved the final manuscript.

## Pre-publication history

The pre-publication history for this paper can be accessed here:

http://www.biomedcentral.com/1471-2318/10/62/prepub

## Supplementary Material

Additional file 1 Supplemental Table S1: Associations between relative telomere length and potential confounders for the two age cohorts Click here for file
