# Supplementary material for: Cognitive performance and leukocyte telomere length in two narrow age-range cohorts: a population study
Source: BMC Geriatr. 2010 Sep 16;10:62. doi: 10.1186/1471-2318-10-62 (PMC2949672; doi:10.1186/1471-2318-10-62)
Supplement: Additional file 1 — Supplemental Table S1: Associations between relative telomere length and potential confounders for the two age cohorts [file 1471-2318-10-62-S1.DOC]

**Additional file 1**

**Supplemental Table S1** Associations between relative telomere length and potential confounders for the two age cohorts

| **Variable** | **40+ Age Cohort** | ***r*** | ***p*-value** | **60+ Age Cohort** | ***r*** | ***p*-value** |
| --- | --- | --- | --- | --- | --- | --- |
| Alcohol consumption^a^ | Cohort | 0.003 | 0.958 | Cohort | -0.155 | 0.008** |
|  | Men | -0.023 | 0.777 | Men | -0.129 | 0.106 |
|  | Women | 0.043 | 0.554 | Women | -0.154 | 0.072 |
| BMI^a^ | Cohort | -0.040 | 0.470 | Cohort | -0.043 | 0.459 |
|  | Men | -0.041 | 0.618 | Men | 0.013 | 0.873 |
|  | Women | -0.033 | 0.657 | Women | -0.085 | 0.323 |
| Physical activity^a^ | Cohort | -0.114 | 0.034* | Cohort | -0.022 | 0.200 |
|  | Men | -0.107 | 0.184 | Men | -0.045 | 0.574 |
|  | Women | -0.106 | 0.141 | Women | 0.017 | 0.848 |
| Sex^b^ |  |  | 0.366 |  |  | 0.200 |
| Smoking^b^ | Cohort |  | 0.098 | Cohort |  | 0.679 |
|  | Men |  | 0.707 | Men |  | 0.070 |
|  | Women |  | 0.081 | Women |  | 0.782 |
| Antioxidant vitamin use^b^ | Cohort |  | 0.963 | Cohort |  | 0.886 |
|  | Men |  | 0.943 | Men |  | 0.795 |
|  | Women |  | 0.937 | Women |  | 0.530 |
| Socioeconomic Status^b^ | Cohort |  | 0.099 | Cohort |  | 0.847 |
|  | Men |  | 0.234 | Men |  | 0.721 |
|  | Women |  | 0.219 | Women |  | 0.897 |
| Systolic blood pressure ^a, c^ | Cohort | 0.092 | 0.087 | Cohort | 0.036 | 0.536 |
|  | Men | 0.015 | 0.856 | Men | 0.053 | 0.510 |
|  | Women | 0.157 | 0.029* | Women | 0.044 | 0.608 |

BMI = body mass index; **p* < 0.05

^a^ Relationships were assessed using Pearson’s correlation coefficients

^b^ Relationships were assessed using independent *t*-tests or ANOVA

^c^ Mean of two blood pressure measurements
